# Pathological Role of Angiotensin II in Severe COVID-19

**DOI:** 10.1055/s-0040-1713678

**Published:** 2020-06-26

**Authors:** Wolfgang Miesbach

**Affiliations:** 1 Department of Haemostaseology and Haemophilia Center, Institute of Transfusion Medicine, Medical Clinic 2, University Hospital Frankfurt, Frankfurt, Germany

**Keywords:** renin–angiotensin system, angiotensin, inflammation, coagulopathy, COVID-19

## Abstract

The activated renin–angiotensin system induces a prothrombotic state resulting from the imbalance between coagulation and fibrinolysis. Angiotensin II is the central effector molecule of the activated renin–angiotensin system and is degraded by the angiotensin-converting enzyme 2 to angiotensin (1–7). The novel coronavirus infection (classified as COVID-19) is caused by the new coronavirus SARS-CoV-2 and is characterized by an exaggerated inflammatory response that can lead to severe manifestations such as acute respiratory distress syndrome, sepsis, and death in a proportion of patients, mostly elderly patients with preexisting comorbidities. SARS-CoV-2 uses the angiotensin-converting enzyme 2 receptor to enter the target cells, resulting in activation of the renin–angiotensin system. After downregulating the angiotensin-converting enzyme 2, the vasoconstrictor angiotensin II is increasingly produced and its counterregulating molecules angiotensin (1–7) reduced. Angiotensin II increases thrombin formation and impairs fibrinolysis. Elevated levels were strongly associated with viral load and lung injury in patients with severe COVID-19. Therefore, the complex clinical picture of patients with severe complications of COVID-19 is triggered by the various effects of highly expressed angiotensin II on vasculopathy, coagulopathy, and inflammation. Future treatment options should focus on blocking the thrombogenic and inflammatory properties of angiotensin II in COVID-19 patients.

## Introduction

Coronavirus disease (COVID-19) is a recent pandemic infection caused by an enveloped, nonsegmented single-stranded ribonucleic acid (RNA)-β coronavirus 2 (SARS-CoV-2). SARS-CoV-2 is the seventh member of the coronavirus 1 that can cause various symptoms ranging from a mild cold to severe respiratory diseases such as severe acute respiratory syndrome (SARS) and Middle Eastern respiratory syndrome (MERS) with mortality rates of 10% for SARS and 37% for MERS. 2

In recent years, two other coronary viral infections have spread and led to severe respiratory diseases: SARS and MERS.

SARS-CoV first appeared 18 years ago. 3 During the SARS epidemic in 2002, more than 8,000 infected patients of all ages and 744 deaths were documented in 26 countries on 5 continents. The main clinical manifestations were upper respiratory symptoms, rapid progression of pneumonia, and approximately 20 to 30% had to be admitted to intensive care. 4 In patients over 65 years of age, the mortality rate was over 50%. Of the patients treated or dying in the intensive care unit, 11.4% developed disseminated intravascular coagulation.

COVID-19 is characterized by an exaggerated inflammatory response that can lead to severe complications, acute respiratory distress syndrome (ARDS), and sepsis shortly after the onset of symptoms. 5 Thrombotic events and coagulopathy have also been described in COVID-19. 6

The transition from mild to severe in patients with COVID-19 can be rapid without predicting symptoms, and older male and obese patients with comorbidities have a higher risk of developing severe symptoms. 7

Acute lung failure is a pathology of many diseases, and a combination of antiviral and anti-inflammatory treatments is recommended for COVID-19. 8 Unfortunately, no specific drug or vaccine has yet been approved for the treatment of human coronavirus.

Therefore, the underlying pathomechanism of COVID-19-induced changes should be investigated to identify specific treatment options.

## Clinical COVID-19 Manifestations

Of the total of 44,672 cases of COVID-19 published by the Chinese Centre for Disease Control and Prevention, 81% had mild symptoms, 14% severe, and 5% critical manifestations. 9 The case fatality rate was 2.3 and 14.8% in patients aged ≥80. Older patients and patients with comorbidities and higher body mass index are more likely to have serious complications of COVID-19. Severe and critical cases suffer from sepsis and ARDS, and coagulopathy occurs in 50% of cases. 10

Sepsis, cytokine storm, and viral bypassing of the cellular immune response have been described in connection with human coronavirus infections 11 12 associated with neutrophilia and pulmonary infiltration of neutrophils and macrophages in respiratory syndromes. 13 14

Pulmonary symptoms and pneumonia are predominant in COVID-19. 15 Pneumonia can be complicated by hypoxic pulmonary vasoconstriction, which is a homeostatic reflex contraction of the pulmonary vascular smooth muscle in response to low regional oxygen partial pressure that redirects blood to more oxygenated lung segments. 16 In a study using remdesivir, a nucleoside analogue drug that inhibits viral RNA polymerases there is a mortality rate of 18% in ventilated COVID-19 patients. 17

Sepsis is the most common cause of acute lung injury and ARDS. 18 ARDS is characterized by diffuse alveolar damage and is often complicated by pulmonary hypertension. 19 In patients with ARDS, a subgroup of ARDS survivors develop a fibroproliferative response characterized by fibroblast accumulation and deposition of collagen and other extracellular matrix components in the lung. The development of severe fibroproliferative lung disease is associated with a poor prognosis with high mortality and/or prolonged ventilator dependence. 20

All patients with severe complications experienced extrapulmonary symptoms and organ injuries. In a multivariable analysis comparing clinical and laboratory parameters of 137 surviving patients from 54 nonsurvivors, death occurred median on the 18th day of hospital treatment after mechanical ventilation for 14.5 days. 10 In three patients an attempt was made to perform extracorporeal membrane oxygenation. All 54 deceased patients developed sepsis (100 vs. 42% of survivors), 53 patients suffered from respiratory failure (98 vs. 36%), 50 patients suffered from ARDS (93 vs. 7%), 28 patients suffered from heart failure (52 vs. 12%), and 38 patients suffered from septic shock (70 vs. 0%). The rate of patients with organ injury was significantly higher in patients with severe outcome. Of 54 patients, 32 patients had a heart injury, 25 had heart failure, and 27 had a kidney injury.

In China, several studies investigated the clinical course of COVID-19, differentiating between severe and nonsevere outcome and collecting data on organ injury. In 6 studies, the clinical course of 1,841 patients was investigated, 21% of whom developed a severe manifestation of the disease. 7 10 12 21 22 23 Median age ranged from 49 to 71.5 years and was up to 28.5 years older than in patients with nonsevere course. Male patients were more affected by severe manifestations from 57.8 to 78%. All patients with severe manifestations had organ injuries, up to 55% heart injuries and up to 50% kidney injuries ( Table 1 ).

**Table 1:** Cardiac and kidney injury and the clinical course of COVID-19

Study	Clinical course	Patients ( *n* )	Age, median	Male sex in severe manifestation (%)	Cardiac injury	Kidney injury
Zhou et al 10	Severe	54	69	70	32	27
	Nonsevere	137	52		1	1
Guan et al 7	Severe	173	52	57.8		5
	Nonsevere	926	45			1
Yang et al 21	Severe	32	64.6	67	9	12
	Nonsevere	20	51.9		3	3
Wang et al 22	Severe	36	66	61.1	8	3
	Nonsevere	102	51		2	2
Huang et al 12	Severe	45	49	73	4	3
	Nonsevere	49	49		1	0
QI et al 23	Severe	50	71.5	78	3	
	Nonsevere	217	43		0	

Angiotensin-converting enzyme 2 (ACE2) is critical for heart function. Angiotensin II (Ang II) infusions in mice resulted in increased blood pressure, myocardial hypertrophy, and fibrosis, whereas these effects could be counteracted with recombinant human ACE2. 24 Loss of ACE2 can further exacerbate cardiac damage. 25

Myocardial infarction appears to be an important complication of the disease, which, according to Chen et al, was also found in the autopsy of a 53-year-old female patient. 5 In a meta-analysis, cardiac troponin I levels are significantly increased in patients with severe COVID-19 infection compared with patients with milder forms of the disease. 26

Cardiac injury has been shown in a cross-sectional study with 150 patients included, 126 mild and 24 severe cases 5 and has been confirmed as common condition among hospitalized patients associated with higher risk of in-hospital mortality. 27 Of a total of 416 patients, 19.7% had cardiac injury and highly significant elevated levels of high-sensitive troponin I levels. The mortality of these patients was significantly higher with 51.2% than those without cardiac injury (4.5%).

Laboratory and imaging findings suggest an increased risk of thrombotic events in patients with COVID-19 infection, but the incidence of thrombosis in patients with COVID-19 has not been determined.

Coagulopathy has been described in up to 50% of severe manifestations of COVID-19. It has been shown that increased D-dimers measured at hospital admission can predict the severe course of COVID-19. 10 D-dimer derives from the cleavage of cross-linked fibrin and reflects both thrombin production and activation of fibrinolysis. Besides the known variability in healthy subjects and its tendency to increase with age, there is an association of increased D-dimer levels and fibrin degradation products under all conditions with an activated coagulation system, such as thrombosis, infection, or malignancy. 28 A large study included 1,099 COVID positive patients from 552 hospitals in China. D-dimer levels above the threshold of 0.5 μg/L were detected in 46.4% of patients; 60% among them developed severe manifestations. In these patients, D-dimer levels were fourfold increased at 2.12 ug/mL (0.77–5.27) compared with nonsevere patients (0.61 ug/mL, 0.35–1.29). Interestingly, none of the nonsevere patients had D-dimer levels increasing more than three times at admission. 7

In a study of 184 critically ill patients with COVID-19 from 3 Dutch hospitals, there was a 31% incidence of thrombosis instead of the use of standard doses of thromboprophylaxis with low molecular weight heparin. The majority of patients suffered from lung embolism, but stroke also occurred in three patients. 29

In addition, microthrombosis was described in a case series of four autopsies of COVID-19 infected patients from New Orleans with sudden respiratory decompensation. The autopsy findings showed small vessels with thromboembolism and small thrombi together with scattered areas of diffuse alveolar damage. These findings usually worsened the course of the disease and could hardly be diagnosed early. The D-dimers found at the time of death were elevated in only two patients, which shows that microthrombosis does not usually lead to generalized coagulation activation. 30

## The Renin–Angiotensin System

The renin–angiotensin system (RAS) was first described more than 40 years ago and has led to a broader understanding of cardiovascular pathophysiology. 31 The RAS is known for its role in the regulation of blood pressure, electrolyte balance, and vascular remodeling. 32 Continuous basic and clinical research has enabled significant advances in therapy and developed the treatment of hypertension and left ventricular dysfunction as well as severe heart failure.

Renin is released by the kidney in response to renal hypoperfusion, reduced sodium intake, and sympathetic activation. A cascade of proteolytic reactions leads to the formation of Ang peptides with different functions. The ACE is a zinc-dependent peptidase responsible for the conversion of Ang I into vasoconstrictive Ang II. ACE2 counteracts the activity of ACE by reducing the amount of Ang II, increasing Ang, 1 2 3 4 5 6 7 and cleaving Ang I to Ang 1 2 3 4 5 6 7 8 9 and thereby attenuating the effects of Ang II. The RAS produces opposite effects and Ang 1 2 3 4 5 6 7 directly antagonizes the effects of Ang II and mediates vasodilatory and antiproliferative effects because it induces the release of nitric oxide and prostaglandin I2 after stimulation of the endothelial AT2 receptor. 33 34

ACE2 is expressed predominantly by vascular endothelial cells of the lung, but also of extrapulmonary tissue, heart, nervous system, intestine, kidneys, blood vessels, and muscles on cell surfaces. 35 36

In addition, ACE2 has functions that are independent of RAS. In vitro and in vivo studies have shown that ACE2 acts as a functional SARS-CoV-2 receptor as well as the transmembrane serine protease TMPRSS2, which is required for host cell entry and subsequent viral replication. 37

Remarkably, the SARS-CoV-2 protein recognizes human ACE2 with an even higher binding affinity than the spike of SARS-CoV. 38 Preclinical studies have shown that after binding of SARS-CoV to its receptor, ACE2 activates RAS leading to downregulation of the expression of ACE2, which in turn results in excessive production of Ang II. 39 40 41 42

Therefore, ACE2 plays a dual role in COVID-19: Initially, it acts as a receptor for SARS-CoV-2 entry, then, in the context of SARS-CoV-2 infection, ACE2 is downregulated, which increases Ang II.

## The Role of Angiotensin II

Ang II is the central effector molecule of activated RAS. Elevated levels of Ang II have been reported mostly in patients with hypertension and heart failure, indicating the benefit of RAS inhibitors. 43

There are two Ang II receptors, type 1 and type 2, which indicate the effect of RAS inhibitors. 43 Most cardiovascular effects of Ang II are attributed to the type 1 receptor.

Notably, Ang II is one of the most potent vasopressors known when linked to its type 1 Ang receptor. The vasopressor effects of Ang II were investigated in a multinational, double-blind, randomized controlled trial (ATHOS-3) in 163 patients with vasodilatory shock. 44 Ang II effectively raised blood pressure in patients with vasodilatory shock who did not respond to high doses of conventional vasopressors. However, a high rate of thrombosis was observed in 12.9% of patients and the use of thromboprophylaxis was recommended. 45 Interestingly, skin problems possibly caused by thrombosis of small vessels were also observed.

There is evidence for the important role of Ang II in vascular cell growth and tissue remodeling after hypertension, vascular injury, heart failure, and atherosclerosis. 46 47

Recently, Ang II has been shown to stimulate the production of pulmonary fibroblast procollagen via the AT1 receptor in lung injury. 48 In addition, Ang II promotes the growth of vascular smooth muscle cells (VSMCs), which induces cellular hypertrophy in the pathology of hypertension and atherosclerosis.

Ang II contributes to endothelial dysfunction, the development of arteriosclerosis, and microvascular thrombosis. Ang II stimulates tissue factor (TF) expression both in vitro and in vivo. 49 TF is the physiological initiator of blood coagulation and as a consequence of its activation, TF becomes dominant over TF pathway inhibitor, resulting in prothrombotic endothelium. In addition, Ang II stimulates platelet-derived growth factor production and increases platelet aggregation. Arterial thrombotic events such as stroke or myocardial infarction are associated with increased platelet aggregation. 50

In addition to that endothelial microparticles which are key players in the pathogenesis of vascular diseases, inflammation, coagulation, and angiogenesis are increased in many age-related vascular diseases such as coronary artery disease, but also stimulated by Ang II. 51

It has also been shown that Ang II infusion leads to abnormal inflammatory and thrombotic reactions in the microcirculation. In hypertensive rats, Ang II accelerates arterial thrombosis of the carotid artery via the AT1 receptor. 52

The prothrombogenic effects are not restricted to large arteries. There is also accelerated microvascular thrombosis in arterioles and to a lesser extent in venules after Ang II infusions. 53

Clinical data also support the role of Ang II in fibrinolysis. Plasminogen activator inhibitor 1 (PAI-1) is the major inhibitor of the fibrinolytic system and elevated levels have been found to be associated with coronary artery disease, deep vein thrombosis, and malignancy. 54 Mature fat cells are an important source of PAI-1, and its expression correlates with visceral fat mass. In vitro and vivo studies have shown that Ang II stimulates the expression and release of PAI-1. Ang II also stimulates the expression of PAI-1 messenger RNA (mRNA) in endothelial cells and increases plasma PAI-1 levels in a dose-dependent manner. The infusion of Ang II in healthy volunteers led to a significant increase in PAI-1 concentrations. 55

In the HEART study, PAI-1 concentrations were significantly lower when administering both ramipril and captopril, 56 both of which can suppress the formation of Ang II in adipose tissue. 54

In addition, inhalation of plasminogen, whose conversion to plasmin is inhibited by PAI-1, improves lung lesions and hypoxemia in patients with COVID-19. 57

There is an association between activated RAS, increased Ang II, and inflammatory cytokine expression and activation. Ang II has proinflammatory properties, including the increase of interleukin (IL)-6, which was demonstrated after infusion of Ang II in healthy controls. 58 Furthermore, Ang II has been shown to induce IL-6 transcription in VSMC. 59 In VSMC, the expression of IL-6 could be induced by Ang II, 60 which may explain the underlying mechanism in the progression of Ang II-induced atherosclerosis.

IL-6 is a multifunctional cytokine that mediates the proliferation of B-lymphocytes during antibody synthesis. Ang II stimulates the release of IL-6, contributing to cytokine storm and poorer outcomes in patients with COVID-19. 59

The function of ACE2 has also been demonstrated in an ACE2 knockout mouse model: The loss of ACE2 expression preceded acute lung damage in various models. ACE2 knockout mice showed more severe lung damage caused by increased hydrostatic pressure, reduced perfusion, and severe pulmonary edema. 41 Administration of recombinant ACE2 protects the lungs from severe lung damage. Consequently, Ang II is directly associated with lung tissue damage and ACE2 is inversely associated with lung tissue damage.

Organ damage to the lung, heart, and kidney is a major cause of severe clinical manifestations in COVID patients. Already in 2005, ACE2 was shown to protect the mouse lungs from ARDS in ACE2 knockout mice. In particular, the injection of SARS-CoV spikes into mice exacerbates acute lung failure in vivo, which can be attenuated by blocking the renin–angiotensin signaling pathway, suggesting that activation of pulmonary RAS influences the pathogenesis of ARDS and SARS. 39 41 In autopsies of patients who died from SARS, 35% of heart samples showed the presence of SARS-CoV associated with reduced ACE2 expression. 61

Remarkably, the unbalanced RAS was found in COVID-19 patients. In an investigation of epidemiological, clinical, laboratory chemical, and radiological characteristics and potential biomarkers to predict disease severity in 2019-nCoV-infected patients in Shenzhen, Ang II levels in the plasma sample of 2019-nCoV-infected patients were shown to be significantly elevated and linearly associated with viral load and lung damage in critically ill patients. 62

Table 2 summarizes the possible effects of Ang II on the clinical symptoms of COVID-19 patients. The complex clinical picture of patients with severe complications of COVID-19 is triggered by the different effects of highly expressed Ang II on vasculopathy, coagulopathy, and inflammation.

**Table 2:** Possible effects of angiotensin II on clinical symptoms of COVID-19

Clinical COVID-19 symptom	Sepsis	ARDS	Organ injury (cardiac, kidney)	Thrombosis
Ang II-induced inflammation	Increase of IL-6 (59–61)			Increase of IL-6 (59–61)
Ang II-induced vasculopathy and thrombosis		Vasoconstriction 16 Increased hydrostatic pressure 41 Fibroproliferation 20 24 48 Vascular smooth muscle cells 60 61	Vasoconstriction 16 Fibroproliferation 20 24 48 Vascular smooth muscle cells 60 61	Vasoconstriction 16 Fibroproliferation 20 24 48 Vascular smooth muscle cells 60 61
Ang II-induced coagulopathy				Increase of TF 49 and PAI-1 54 55 Increased platelet aggregation 50 67 and increase of PDGF 50

Abbreviations: ANG II, angiotensin II; ARDS, acute respiratory distress syndrome; IL-6, interleukin-6; PAI-1, plasminogen activator inhibitor 1; PDGF, platelet-derived growth factor; TF, tissue factor.

## Treatment Options of Angiotension 2-Related Clinical Symptoms

It could be shown that patients with COVID-19 have an unbalanced RAS with highly expressed Ang levels. In addition, the presence of comorbidities such as arterial hypertension is significantly associated with a worse outcome. Of a total of 1,430 patients in 3 studies, hypertension was common in up to 48% of patients and there were significantly more severe manifestations in 36 to 48% of patients compared with severe manifestations in 14 to 24% of patients without hypertension ( Table 3 ).

**Table 3:** Frequency of hypertension in COVID-91 patients and clinical outcome

Study	Patients ( *n* )	Patients with hypertension (%)	Worse outcome of patients with hypertension versus of patients with no hypertension
Guan et al 7	1,099	15	36 vs. 14%
Zhou et al 10	191	30	48 vs. 23%
Zhang et al 11	140	30	38 vs. 24%

The presence of hypertension is associated with significantly higher mortality. The odds ratio for mortality in hypertension is between 1.70 (0.92–3.13) 12 and 3.05 (1.6 - 5.9). 10 However, the data were not adjusted to other risk factors and it became not clear from the studies whether the hypertension was previously known or was only diagnosed during the hospital stay and whether treatment, for example, with RAS-inhibiting drugs, was administered.

Therefore, RAS-inhibiting drugs, such as ACE inhibitors (ACEIs) or angiotensin receptor blockers (ARBs), may be used in COVID-19-infected patients to treat hypertension and other Ang II-related complications. A preclinical study showed that ARB drugs, especially losartan, are effective in relieving acute lung injury caused by SARS-CoV in mice. 62

However, ACEI and ARB have shown an increase in ACE2 expression (in heart tissue) in animal models. 63 There is concern that a possible higher expression of pulmonary membrane-bound ACE2 (which has not been confirmed to date) may lead to easier entry of the virus into respiratory cells when RAS-inhibiting drugs are taken.

On the other hand, both ACEI (lisinopril) and ARB (losartan) can upregulate cardiac ACE2 mRNA by a factor of 3 to 4. In an animal model, the ACEI enalapril attenuated the downregulation of cardiac ACE2 after myocardial infarction. 64 65

There is a clear benefit of ACEI and ARB in reducing thrombotic effects in hypertensive patients. 66 67 Protection against severe lung damage could also be achieved by administering recombinant ACE2. ACE2 knockout mice showed more severe lung damage caused by increased hydrostatic pressure, reduced perfusion, and severe pulmonary edema. 41 Administration of recombinant ACE2 protects the lungs from severe lung damage. Consequently, Ang II is directly associated with lung tissue damage and ACE2 is inversely associated with lung tissue damage.

Recently, it could be shown that human recombinant soluble ACE2 can block early stages of SARS-CoV-2 infections 68 and could in the future also be used to treat COVID-19 patients, which is currently being tested in a phase 2 clinical trial.

Currently, numerous drugs are studied in clinical trials. A small cohort of critically ill patients was treated with remdesivir, a nucleoside analogue that inhibits viral RNA polymerases, and a clinical improvement was observed in 36 of 53 patients (68%). 17 Randomized controlled trials are ongoing and will soon provide more evidence of the safety and efficacy of remdesivir for Covid-19.

Finally, considering COVID-19 as a procoagulant disease, prophylactic anticoagulation with low molecular weight heparin should be initiated as soon as possible to prevent thrombotic events and to counteract the proinflammatory influence of cytokines. 69

## Conclusion

The transition from mild to severe symptoms can occur rapidly in patients with COVID-19 without predicting signals, and older male and obese patients with comorbidities have a higher risk of developing severe symptoms.

The RAS plays an important role in COVID-19. ACE2 acts as functional SARS-CoV-2 receptor, which leads to a downregulation of ACE2 and a higher expression of Ang II. ACE2 is predominantly expressed by vascular endothelial cells of the lung, but also in extrapulmonary tissue, heart, nervous system, intestine, kidneys, blood vessels, and muscles on cell surfaces, which may explain the multiorgan dysfunction observed in patients with COVID-19.

The complex clinical picture of patients with severe complications of COVID-19 includes pneumonia, ARDS, sepsis, coagulopathy, high rate of thrombosis, and organ damage and is triggered by various effects of highly expressed Ang II on vasculopathy, coagulopathy, and inflammation.

The combination of numerous pathophysiological changes caused by Ang II in COVID-19 could explain the rapid development of severe patients and why older and obese patients are particularly affected.

Future treatment options should focus on blocking the thrombogenic and inflammatory properties of Ang II in COVID-19 patients.
